# Supplementary figures and images for: De novo identification of toxicants that cause irreparable damage to parasitic nematode intestinal cells
Source: PLoS Negl Trop Dis. 2020 May 26;14(5):e0007942. doi: 10.1371/journal.pntd.0007942 (PMC7274465; doi:10.1371/journal.pntd.0007942)

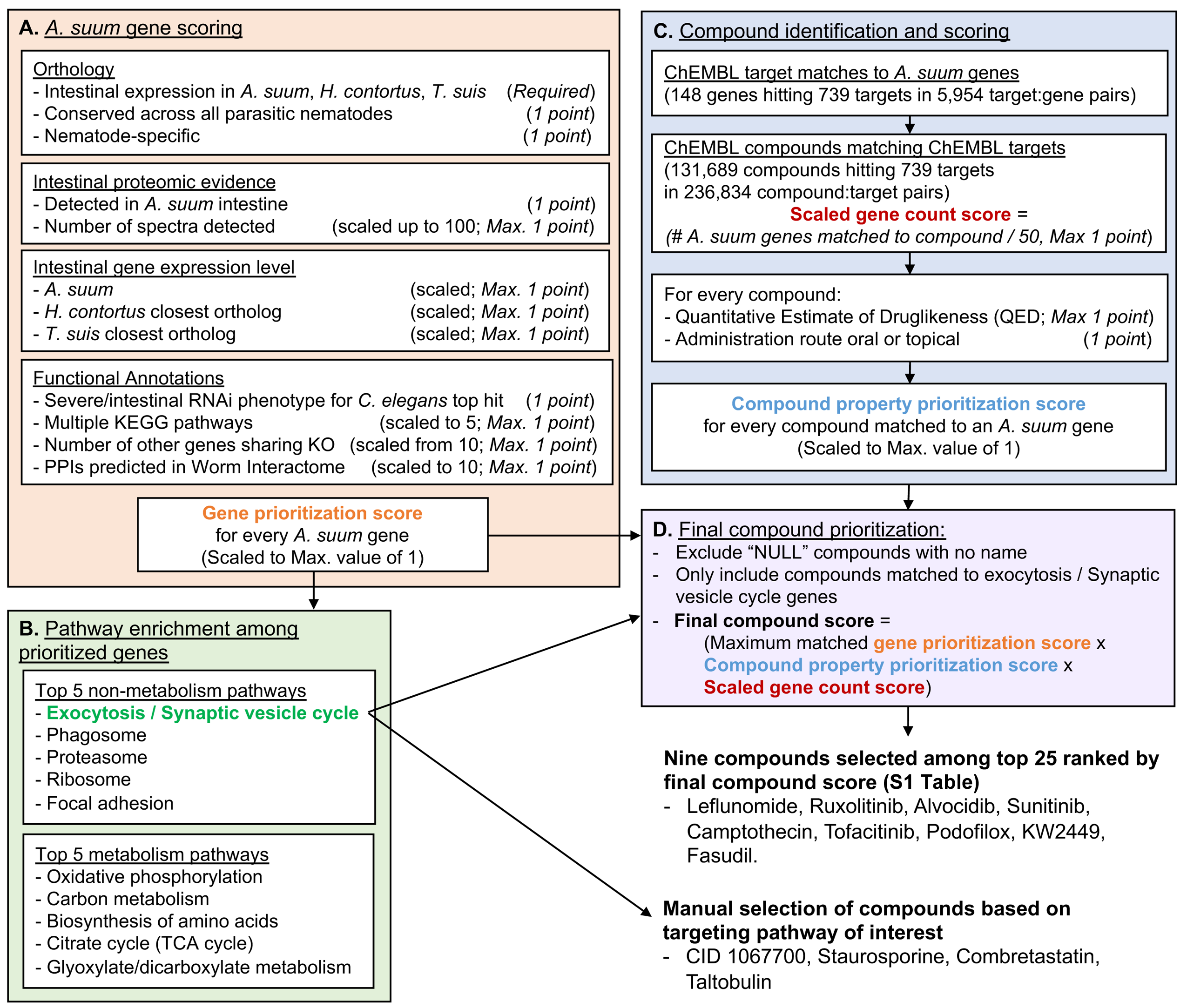

Supplement: S1 Fig — Major steps of the prioritization include (A) A. suum gene scoring, (B) Pathway enrichment among prioritized genes, (C) Compound identification and scoring and (D) Final compound prioritization. (TIF) [file pntd.0007942.s001.tif]

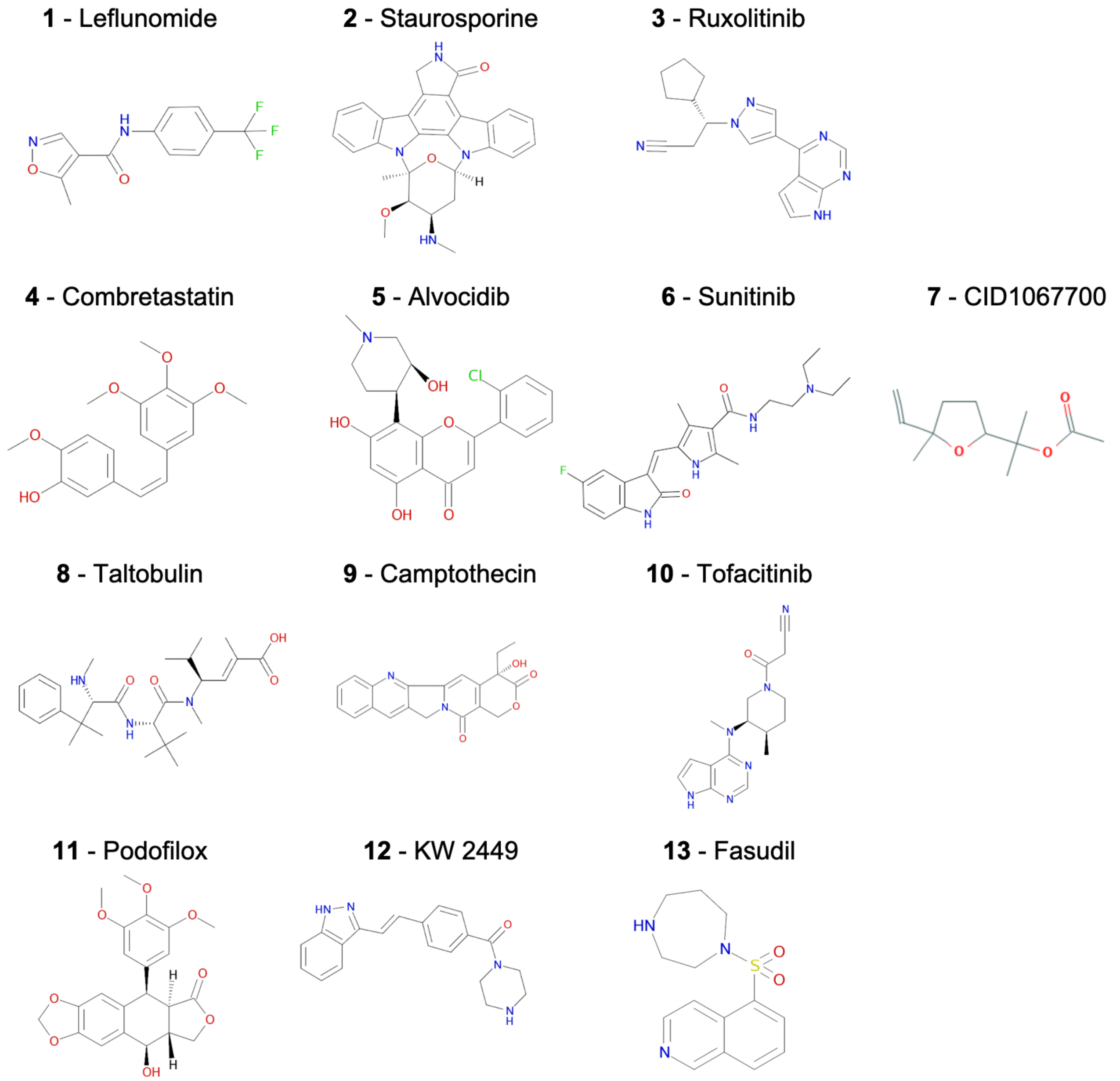

Supplement: S2 Fig — (TIF) [file pntd.0007942.s002.tif]

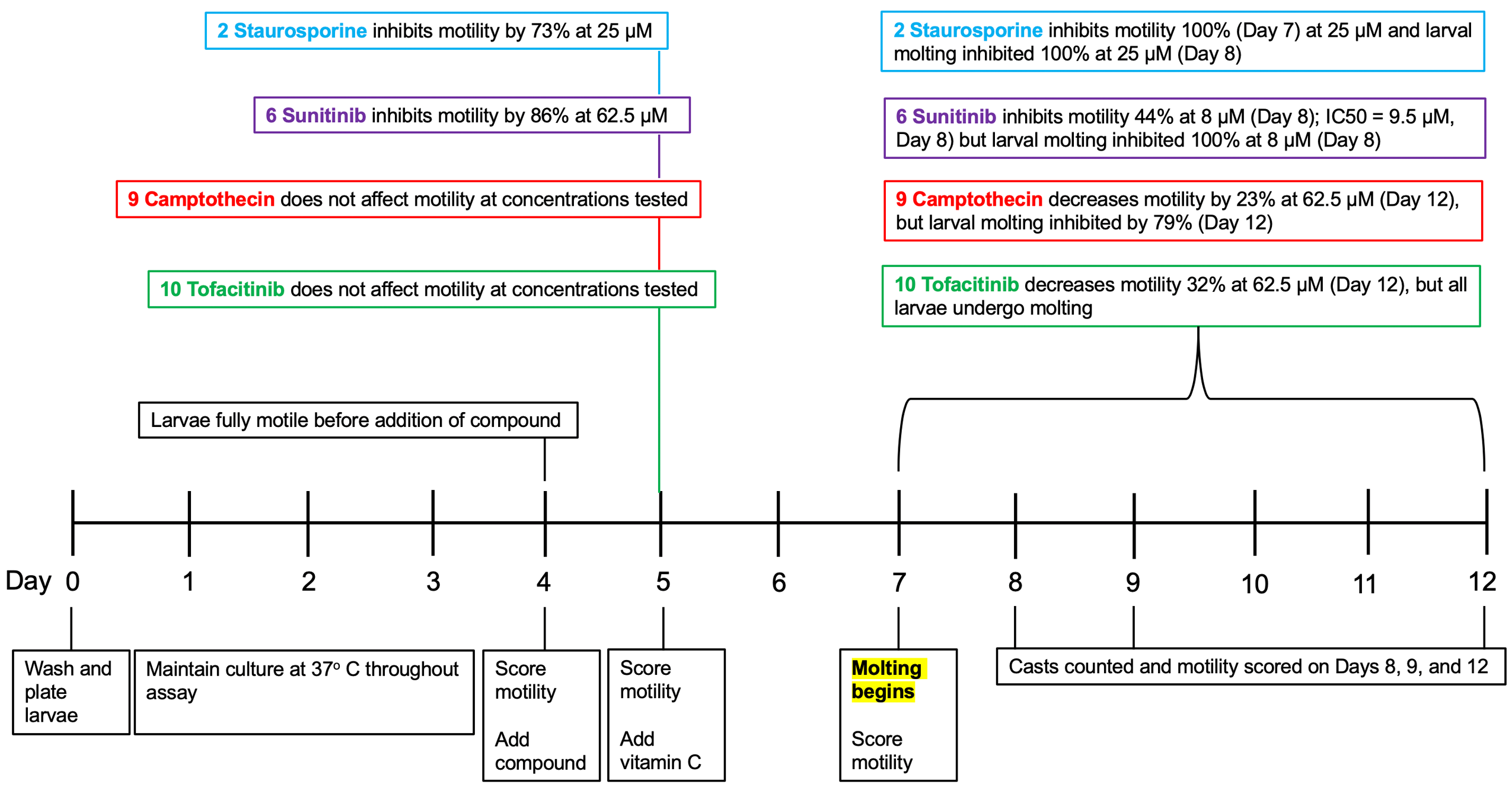

Supplement: S3 Fig — (TIF) [file pntd.0007942.s003.tif]

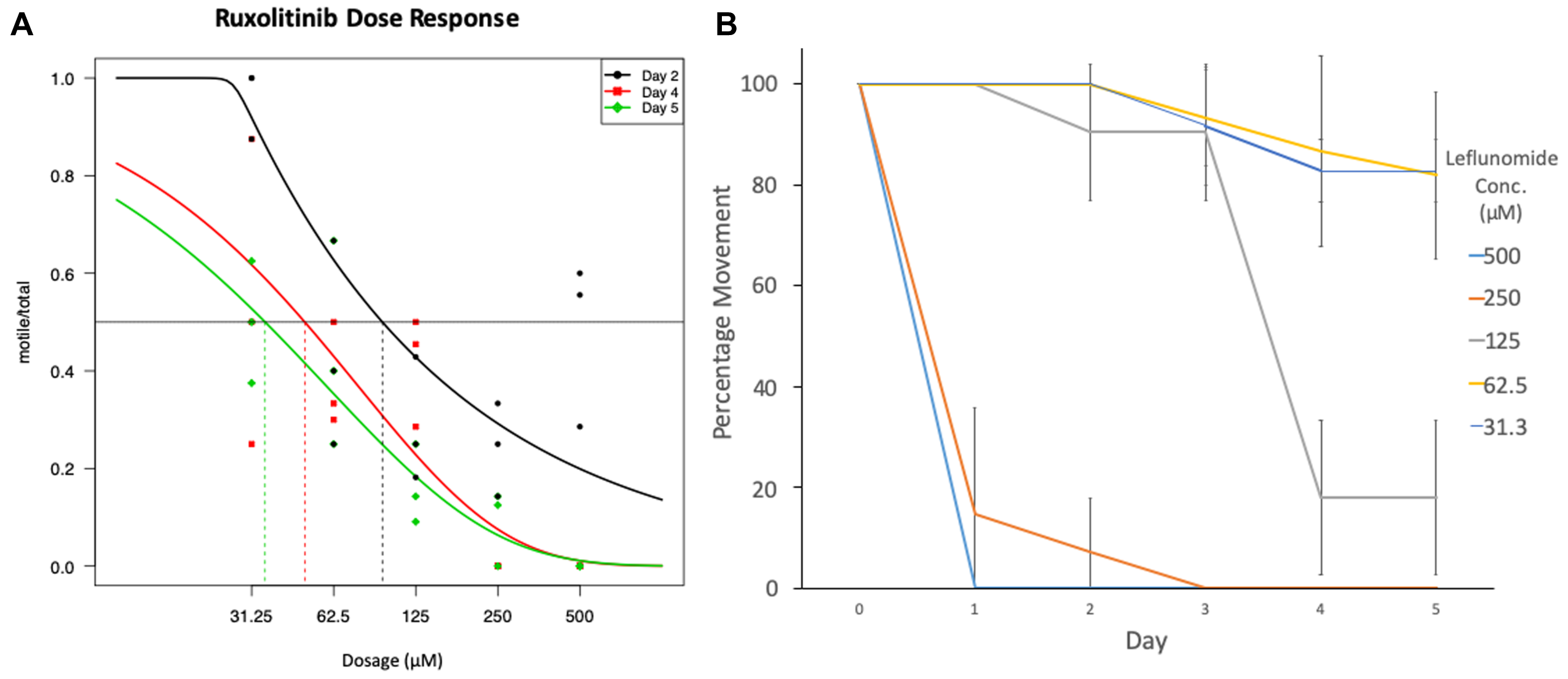

Supplement: S4 Fig — (A) An example to illustrate a lack of fit of dose-response curve. Ruxolitinib Day 2 data (black) showed significant lack of fit for A. suum L3 larvae, primarily due to anomalously high motility of the 500 μM dosage samples. Data for Days 4 and 5 (red and green, respectively) showed good fit. (B) L3 Motility curves for Leflunomide (1). There is rapid inhibition of motility for 250 and 500 μM dosage, but concentrations below 125 μM show delayed inhibition, resembling effects of Sunitinib (6) and Tofacitinib (10) on L3. (TIF) [file pntd.0007942.s004.tif]

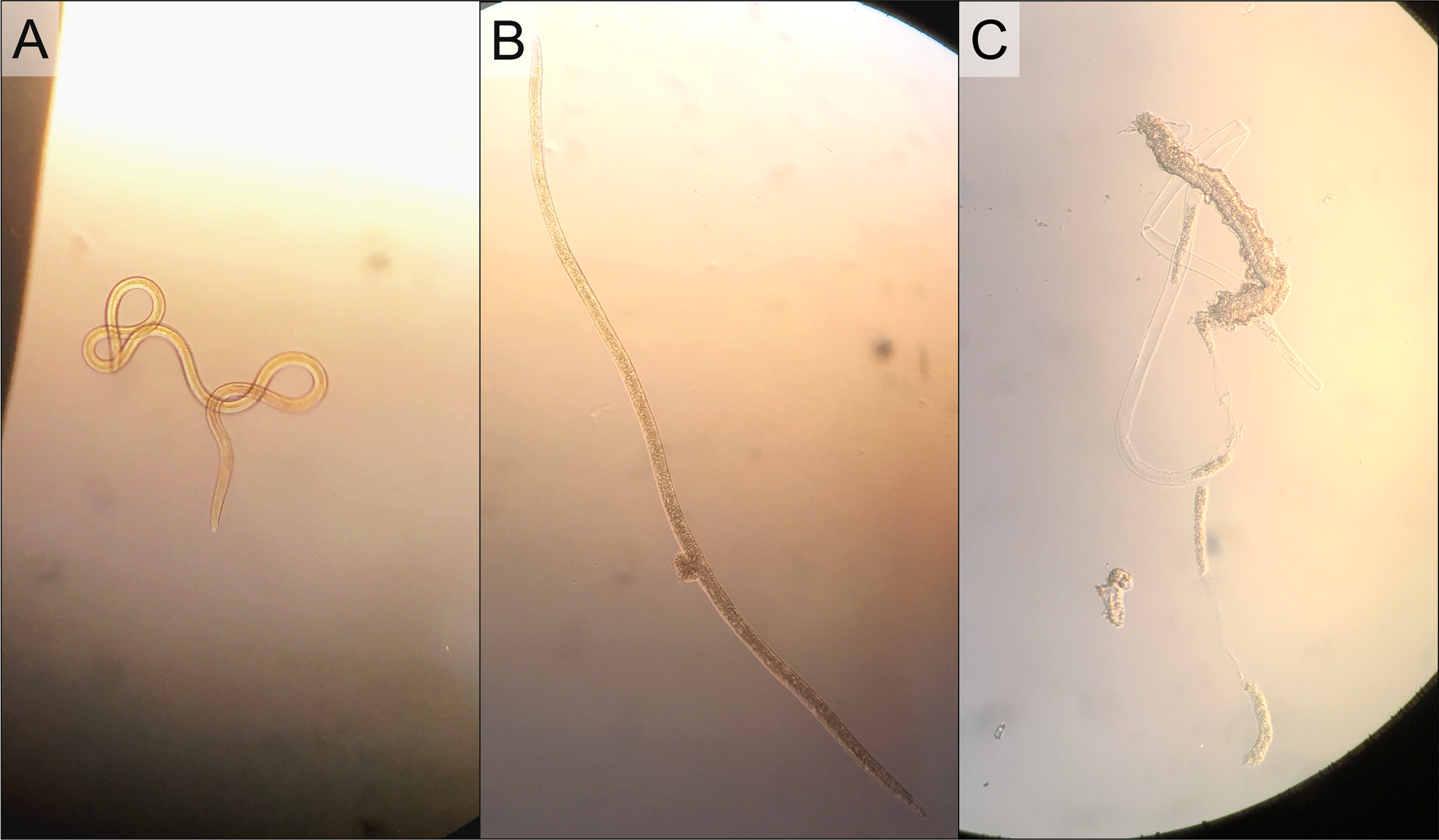

Supplement: S5 Fig — (A) DMSO control L4 larvae, showing a successful molt. (B) Larvae with a bump/protrusion, observed in several of the larvae treated with 16 μM Sunitinib. (C) Example of an L3 that has failed to molt. This phenotype occurred in both treated and DMSO controls that fail to molt. (TIF) [file pntd.0007942.s005.tif]

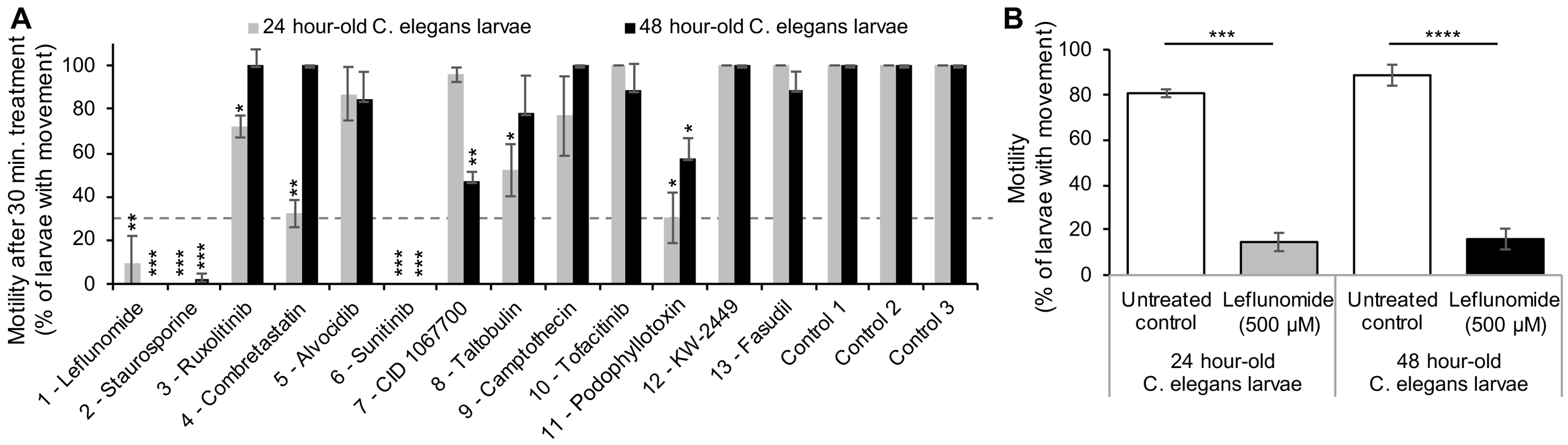

Supplement: S6 Fig — Treatment responses for (A) all 13 inhibitors (1mM, except for Staurosporine at 100 μM), and motility was assessed after 48 hours of treatment. (B) 500 μM Leflunomide treatment and motility was assessed after 30 minutes of treatment. * P ≤ 0.05, ** P ≤ 0.01, *** P ≤ 10−3, *** P ≤ 10−4. P values represent results from a two-tailed T-test (unequal variance). (TIF) [file pntd.0007942.s006.tif]
