# Supplementary material for: De novo identification of toxicants that cause irreparable damage to parasitic nematode intestinal cells
Source: PLoS Negl Trop Dis. 2020 May 26;14(5):e0007942. doi: 10.1371/journal.pntd.0007942 (PMC7274465; doi:10.1371/journal.pntd.0007942)
Supplement: S1 Table — Tested inhibitors are indicated with an asterisk. (DOCX) [file pntd.0007942.s007.docx]

**S1 Table**. The top 25 scored inhibitors. Tested inhibitors are indicated with an asterisk.

| Inhibitor preferred name | Inhibitor ID | Inhibitor prioritization rank | Final Inhibitor Prioritization Score | Inhibitor score | Max. gene prioritization score | Scaled gene count score |
| --- | --- | --- | --- | --- | --- | --- |
| *Sunitinib | CHEMBL535 | 1 | 0.71 | 0.84 | 0.85 | 1 |
| Colchicine | CHEMBL107 | 2 | 0.67 | 0.94 | 0.85 | 0.84 |
| *Podofilox | CHEMBL61 | 3 | 0.66 | 0.91 | 0.85 | 0.86 |
| *Ruxolitinib | CHEMBL1789941 | 4 | 0.65 | 0.93 | 0.85 | 0.82 |
| *Tofacitinib | CHEMBL221959 | 5 | 0.62 | 0.99 | 0.85 | 0.74 |
| *Leflunomide | CHEMBL960 | 6 | 0.51 | 0.99 | 0.85 | 0.6 |
| Riluzole | CHEMBL744 | 7 | 0.50 | 0.96 | 0.85 | 0.62 |
| Paroxetine Hydrochloride | CHEMBL1708 | 8 | 0.49 | 1.00 | 0.85 | 0.58 |
| Palbociclib | CHEMBL189963 | 9 | 0.48 | 0.87 | 0.78 | 0.72 |
| Oxymetazoline Hydrochloride | CHEMBL1200791 | 10 | 0.48 | 0.97 | 0.85 | 0.58 |
| Nabumetone | CHEMBL1070 | 11 | 0.47 | 0.93 | 0.85 | 0.6 |
| Sulindac | CHEMBL15770 | 12 | 0.47 | 0.98 | 0.85 | 0.56 |
| Modafinil | CHEMBL1373 | 13 | 0.47 | 0.98 | 0.85 | 0.56 |
| Olanzapine | CHEMBL715 | 14 | 0.46 | 0.97 | 0.85 | 0.56 |
| Promethazine Hydrochloride | CHEMBL1200750 | 15 | 0.45 | 0.95 | 0.85 | 0.56 |
| Albendazole | CHEMBL1483 | 16 | 0.44 | 0.93 | 0.85 | 0.56 |
| Carbamazepine | CHEMBL108 | 17 | 0.43 | 0.90 | 0.85 | 0.56 |
| *Fasudil | CHEMBL38380 | 18 | 0.35 | 0.44 | 0.85 | 0.94 |
| *KW-2449 | CHEMBL1908397 | 19 | 0.34 | 0.40 | 0.85 | 1 |
| *Alvocidib | CHEMBL428690 | 20 | 0.31 | 0.37 | 0.85 | 0.98 |
| Acacetin | CHEMBL243664 | 21 | 0.30 | 0.46 | 0.85 | 0.78 |
| Biochanin | CHEMBL131921 | 22 | 0.30 | 0.46 | 0.85 | 0.78 |
| *Camptothecin | CHEMBL65 | 23 | 0.28 | 0.41 | 0.85 | 0.82 |
| Indirubin Sulfate | CHEMBL258805 | 24 | 0.28 | 0.40 | 0.85 | 0.84 |
| Bms-387032 | CHEMBL296468 | 25 | 0.26 | 0.38 | 0.85 | 0.8 |
