# Supplementary material for: De novo identification of toxicants that cause irreparable damage to parasitic nematode intestinal cells
Source: PLoS Negl Trop Dis. 2020 May 26;14(5):e0007942. doi: 10.1371/journal.pntd.0007942 (PMC7274465; doi:10.1371/journal.pntd.0007942)
Supplement: S5 Table — (DOCX) [file pntd.0007942.s011.docx]

**S5** **Table.** Selected characteristics of the thirteen experimentally tested inhibitors.

| Drug | CHEMBL ID | Drug class | Drug subclass | Pass toxicity threshold | Approved drug | Literature target |
| --- | --- | --- | --- | --- | --- | --- |
| **1** Leflunomide | CHEMBL960 | Benzene* | Anilides | Yes | Yes | DHODH/AHR/PTK2B^92-95^ |
| **2** Staurosporine | CHEMBL388978 | Indoles* | Carbazoles | Yes | No | PKC/broad kinase^96^ |
| **3** Ruxolitinib | CHEMBL1789941 | Pyrrolopyrimidines | Pyrrolo[2,3-d]pyrimidines | Yes | Yes | JAK1/JAK2^97,98^ |
| **4** Combretastatin | CHEMBL67 | Stilbenes | Stilbenes | Yes | No | Tubulin^99^ |
| **5** Alvocidib | CHEMBL428690 | Flavonoids | Flavones | Yes | Phase III | CDKs^92^ |
| **6** Sunitinib | CHEMBL535 | Indoles* | Indolines | Yes | Yes | PDGFR/FLT^100^ |
| **7** CID 1067700 | - | Thienopyrans | Thienopyrans | Yes | No | Rab/Ras/Rho GTPases^101^ |
| **8** Taltobulin | CHEMBL182319 | Carboxylic acids* | Amino acids, peptides, and analogues | Yes | No | Tubulin^102^ |
| **9** Camptothecin | CHEMBL65 | Camptothecins | Camptothecins | Yes | No | DNA topoisomerase 1^103^ |
| **10** Tofacitinib | CHEMBL221959 | Piperidines | N-acylpiperidines | Yes | Yes | JAK1/JAK2/JAK3^31^ |
| **11** Podofilox | CHEMBL61 | Lignan lactones | Podophyllotoxins | No | Yes | Tubulin^104,105^ |
| **12** KW2449 | CHEMBL1908397 | Benzopyrazoles | Indazoles | No | Phase I | Serine/Threonine kinases^106^ |
| **13** Fasudil | CHEMBL38380 | Isoquinolines* | Isoquinolines* | No | Phase III | Serotinin receptor / CDKs^107^ |
| * and derivatives |  |  |  |  |  |  |
